# First Case of Neuroinvasive Oropouche Virus in Wisconsin: A Case Report

**DOI:** 10.1093/ofid/ofaf402

**Published:** 2025-07-10

**Authors:** Caroline B Ewing, Dawd Siraj, Alexander Lepak

**Affiliations:** Division of Infectious Disease, Department of Medicine, University of Wisconsin, Madison, Wisconsin, USA; Division of Infectious Disease, Department of Medicine, University of Wisconsin, Madison, Wisconsin, USA; Division of Infectious Disease, Department of Medicine, University of Wisconsin, Madison, Wisconsin, USA

**Keywords:** neuroinvasive virus, Oropouche virus, OROV, reemerging virus, sloth fever

## Abstract

Per the Pan American Health Organization/World Health Organization, Oropouche virus (OROV) is a reemerging arbovirus that poses a yet undetermined degree of threat to the United States, with outbreaks reported in the Caribbean and South America, including new areas of spread, since the end of 2023 and, at this time, there exists no vaccine or disease-specific treatment. Here we describe the presentation and diagnosis of a neuroinvasive case of OROV, the first documented case of OROV in Wisconsin, the first documented case in the United States in 2025, and the first documented US case associated with travel to Panama.

Human and environmental elements have influenced and encouraged the spread of Oropouche virus (OROV) [1]. Changing climates with higher temperatures and more rainfall and the expansion of cities into newly deforested areas may have increased the interaction between vectors and humans [1]. International travel, pertinent to the case we report here, has the potential to expand areas in which OROV is endemic. Given that there are no disease-specific therapies, it is especially important to recognize the risk of spread to new regions and to be cognizant of travel histories and clinical syndromes consistent with OROV, including cases of neuroinvasive disease such as ours.

## CASE REPORT

A 19-year-old immunocompetent woman with recent return from a week-long trip to rural Darién Province in Panama presented to our hospital in Madison, Wisconsin, with recrudescence of meningitis symptoms in the form of a positional headache associated with neck and spine stiffness and pain with associated blurry vision, dizziness, lightheadedness, and unsteadiness. Although she did not have close contact with sick contacts during the trip, she did report a recent hepatitis A outbreak at her school and sick influenza contacts at school. She reported mosquito bites during her trip but otherwise did not report animal contacts.

The patient traveled to rural Darién Province for a week-long service trip in January 2025. During her trip, she sustained 3 mosquito bites. On day 1, the day after her return to Madison, she experienced fever (self-recorded to 103°F), malaise, and diarrhea. She attributed these symptoms to drinking unsanitary tap water on her last day of travel before returning to the United States, and the symptoms abated within 4–5 days. On day 12, she experienced severe, positional headache with associated blurry vision, dizziness, and lightheadedness. These symptoms persisted, despite over-the-counter nonsteroidal anti-inflammatory drug therapy, prompting presentation to a student health center on both days 18 and 21. Examination did not reveal any concerning neurological signs or symptoms. Laboratory tests demonstrated mild leukocytosis (to a white blood cell count of 11.8 × 10^3^/μL), the blood parasite smear and screen were negative, and dengue fever serology was ordered and returned negative results (see Table 1 for a summary of selected laboratory findings). The student health center contacted our infectious disease service, and additional testing was suggested, which required the patient to present to our emergency department (ED) for expedited testing.

**Table 1.** Selected Study Results

Laboratory Investigation	Value or Result			
	Reference Range	d 19	d 22	d 34–37
Blood				
WBC count, ×10^3^/μL	3.8–10.5	11.8	8.1	7.1–8.5
Absolute neutrophil count	1700–7500	8320	5370	3570–4200
β-HCG, total, mIU/mL	≤5.0	…	<2.4	<2.4
Blood parasite smear	Negative	Negative	…	…
Blood parasite screen	Negative	Negative	…	…
CHIKV PCR	No RNA detected	…	…	No RNA detected
CHIKV IgM Ab IV	Negative, ≤0.79; equivocal, 0.80–1.09; positive, ≥1.10	…	3.78	…
Dengue virus PCR	No RNA detected	…	…	No RNA detected
Dengue fever IgG Ab IV	Negative, ≤1.64; equivocal, 1.65–2.84; positive, ≥2.85	0.87	…	…
Dengue fever IgM Ab IV	Negative, ≤1.64; equivocal, 1.65–2.84; positive, ≥2.85	1.10	…	…
Murine typhus	No antibody detected (<1:64)	…	…	<1:64
Rocky mountain spotted fever	No antibody detected (<1:64)	…	…	<1:64
Zika virus PCR	No RNA detected	…	…	No RNA detected
Zika virus IgM Ab	Negative	…	…	Negative
Two sets of peripheral cultures	No growth	…	No growth	…
CSF				
Nucleated cell count, cells/μL	≤5	…	…	72
Lymphocytes, %	…	…	…	97
Glucose, mg/dL	40–80	…	…	51
Protein, mg/dL	15–40	…	…	58
Lactate, mmol/L	1.2–2.6	…	…	1.9
Gram stain	No organisms	…	…	No organisms
Culture	No growth	…	…	No growth
Meningitis/encephalitis pathogen PCR panel				
*E. coli* K1	ND	…	…	ND
*Haemophilus influenzae*	ND	…	…	ND
*Listeria monocytogenes*	ND	…	…	ND
*Neisseria meningitidis*	ND	…	…	ND
*Streptococcus agalactiae*	ND	…	…	ND
*Streptococcus pneumoniae*	ND	…	…	ND
CMV (qualitative)	ND	…	…	ND
Enterovirus	ND	…	…	ND
HSV-1	ND	…	…	ND
HSV- 2	ND	…	…	ND
HHV-6	ND	…	…	ND
Human parechovirus	ND	…	…	ND
VZV	ND	…	…	ND
*Cryptococcus neoformans/gattii*	ND	…	…	ND
Urine				
Zika virus PCR	No RNA detected	…	…	No RNA detected
PRNT titer				
CHIKV PRNT	Negative, <1:10; positive, ≥1:10	…	…	<1:10
OROV PRNT	Negative, <1:10; positive, ≥1:10	…	…	1:2560

Abbreviations: Ab, antibody; CHIKV, chikungunya virus; CMV, cytomegalovirus; CSF, cerebrospinal fluid; HCG, human chorionic gonadotropin; HHV, human herpesvirus; HSV, herpes simplex virus; IV, index value; ND, not detected; OROV, Oropouche virus; PCR, polymerase reaction chain; PRNT, plaque reduction neutralization test; VZV, varicella zoster virus; WBC, white blood cell.

On presentation to the ED (day 22), the patient was experiencing headaches, shaking, weakness, lethargy, and unsteadiness. She was afebrile, tachycardic (pulse rate, 127/min), and otherwise hemodynamically stable. Per the ED physician, her physical examination findings were reassuring against encephalitis or meningitis. The patient's leukocytosis had resolved. The pregnancy test result was negative (Table 1). Two sets of peripheral blood cultures showed no growth. A blood chikungunya virus (CHIKV) immunoglobulin (Ig) M antibody was obtained, with a positive result 5 days later, on day 27 (Table 1). Symptoms improved after the ED visit and completely resolved by approximately day 25. However, the patient had a recurrence of severe headache, fever, neck stiffness, blurry vision, dizziness, weakness, and unsteadiness on day 32. Two days later (day 34), she presented again to the ED due to this recurrence of severe symptoms, at which time she was afebrile, tachycardic (pulse rate, 111/min), and otherwise hemodynamically stable. Results of a complete blood cell count with differential, basic metabolic panel, and liver function tests were unremarkable (Table 1).

Lumbar puncture revealed an elevated nucleated cell count of 72/μL with manual differential demonstrating 97% lymphocytes. Cerebrospinal fluid (CSF) glucose was within normal limits at 51 mg/dL, protein was elevated to 58 mg/dL, and lactate was within normal limits at 1.9 mmol/L. Results of a meningitis/encephalitis pathogen polymerase chain reaction (PCR) panel were negative. Table 1 shows the relevant CSF laboratory findings and the full panel of pathogens tested. The patient was given 2 L of fluids and empiric acyclovir, ceftriaxone, vancomycin, and dexamethasone and was admitted to a medical service.

On admission, our infectious disease service was consulted. The patient was afebrile and hemodynamically stable. Examination findings were notable for painful neck flexion. We recommended stopping empiric acyclovir given mild to moderate symptoms, no seizures, and overall low clinical suspicion for herpes viral encephalitis with supporting negative CSF PCR results for herpes virus simplex 1 and 2. Given the patient's history of travel, symptoms, and positive blood CHIKV IgM antibody, her presentation was thought to be due to aseptic meningitis secondary to CHIKV infection. However, given the difficult to explain biphasic nature of the headaches and meningitis symptoms, we believed that additional testing was warranted for a potential alternative pathogen or coinfection. Additional testing included serum CHIKV, dengue virus, and Zika virus RNA PCR and spotted fever group rickettsial serology (Table 1). These results were all negative, as was the urine Zika virus PCR (Table 1).

During her hospitalization, the patient continued to have headache, fatigue, and neck stiffness; however, with symptomatic management these all resolved before discharge. She remained afebrile throughout the remainder of her hospitalization. Empiric antibiotics were stopped and, after 4 days in the hospital, the patient was discharged home in stable condition. We saw her in the clinic 12 days after discharge from the hospital, at which time she felt well without recurrence of symptoms.

The Wisconsin State Laboratory of Hygiene sent the blood sample, based on the patient's initial positive IgM antibody result for CHIKV, to the Arboviral Diseases Branch at CDC (Centers for Disease Control and Prevention) for confirmation testing. Confirmation testing was performed using a plaque reduction neutralization test (PRNT), with a negative CHIKV but overwhelmingly positive Oropouche virus (OROV) PRNT result (Table 1). This case report did not include factors necessitating patient consent.

## DISCUSSION

Here, we present a case of neuroinvasive OROV: the first documented case of OROV imported to the United States from Panama, the first documented case of OROV in the United States in 2025, and the first documented case of OROV in the state of Wisconsin. According to preliminary data from ArboNET, a surveillance system run by the CDC and health departments, 109 total cases of OROV disease have been identified in the United States, all imported and none known to be domestically acquired, including 2 total cases of neuroinvasive disease (including ours), spanning 7 states, with 103 (94%) identified in Florida [2].

Regarding the patient's positive serum CHIKV IgM antibody result, which led us to initially attribute her presentation to aseptic meningitis secondary to CHIKV infection, an IgM antibody is, by its nature, representative of a patient's acute-phase nonspecific immune reaction. This lack of specificity, which can produce cross-reactivity among IgM antibody tests for several pathogens, is not uncommon. For example, a study of 340 samples collected in a dengue virus–endemic area demonstrated 25.3% specificity of a CHIKV IgM antibody test, which was attributed to cross-reactivity with dengue virus [3]. Given the negative serum CHIKV RNA PCR result (though this was collected later in the patient’s course and would be expected to have higher sensitivity earlier in the course during active replication) and negative serum CHIKV PRNT and the positive serum OROV PRNT results, which were revealed later, her positive serum CHIKV IgM antibody was most likely due to a nonspecific cross-reactivity. Per the CDC, a positive CHIKV IgM antibody needs to be followed by confirmation via PRNT [4] . Given that CHIKV is of the *Alphavirus* genus and OROV is of the *Orthobunyavirus* genus, there is no significant risk of cross-reaction regarding PRNT [2, 4, 5].

OROV is in the Simbu serogroup of the *Orthobunyavirus* genus of the *Peribunyaviridae* family [2, 5]. It is an arbovirus that was first identified in 1955 in the blood of a charcoal burner, who worked in the Melajo forest in Trinidad, after presenting with fever; the virus was so named for the region where the patient lived [6]. Since then, OROV has been identified in Central America, South America, and the Caribbean [2]. The disease is spread to humans primarily by flies called biting midges, *Culicoides paraensis*, sometimes referred to as “no-see-ums” [2]. These flies are present in the United States, including in parts of the Midwest and Southeast, and are more likely to be found in the woods, under decaying vegetation and in the holes of trees, as well as in moss-covered, damp, or muddy areas [2 ]. OROV can also be spread by mosquitoes, including *Aedes serratus*, *Coquillettidia venezuelensis*, and *Culex quinquefasciatus* mosquitoes, which also sometimes act as vectors for the St. Louis encephalitis virus and the West Nile virus [2].

Replication-competent OROV was recently isolated from semen 16 days after the onset of symptoms in an Italian man who had recently traveled to Cuba, indicating the possibility of sexual transmission [7]. Regarding the risk of vertical transmission, our patient's pregnancy test was negative. It is important to test for pregnancy in suspected cases as vertical transmission is possible and associated with congenital abnormalities and fetal death [2]. To our knowledge, our patient represents the first reported case of OROV infection borne from Panama to the United States.

Our case illustrates the classic, biphasic illness of OROV with neuroinvasive disease. The patient's symptoms began with fever, malaise, and diarrhea and transitioned to headache, blurry vision, dizziness, lightheadedness, shaking, weakness, lethargy, and unsteadiness. Her symptoms improved significantly and then, a week later, worsened with fever and meningitis symptoms. Symptoms usually last up to 1 week, but, in as many as 60% of cases, can recur with similar severity from a few days to weeks later [2]. Neuroinvasive disease is uncommon; per the CDC, only as many as 4% of patients with a febrile illness associated with OROV infection will go on to experience neuroinvasive disease [2]. Our patient's later symptoms and CSF with lymphocytic elevated nucleated cell count, elevated protein, and normal glucose level, paired with a negative meningitis/encephalitis pathogen PCR panel, support a diagnosis of neuroinvasive OROV infection. Fortunately, patients tend to recover completely without sequelae over the long term, even after severe disease [2]. Among patients with OROV infection, very low mortality rates have been reported [2].

OROV has been recognized as a reemerging arbovirus that causes significant disease without current pathogen-directed therapies available [8]. Here, we reported on its spread to the midwestern United States via Panama and described its neurological manifestations, associated laboratory findings, and probable associated cross-reactivity with blood CHIKV IgM antibody testing. Per CDC updated interim guidance, from day 0-7 following symptom onset, reverse transcription-PCR (RT-PCR) should be obtained; from day 6-7 following symptom onset, PRNT should be conducted only if the initial RT-PCR test is negative; and from beyond day 7 following symptom onset, PRNT alone should be obtained [2]. PRNT is the preferred test for CSF samples and in the case of recurrence of symptoms, given the potential for viral clearance from the blood prior to testing [9].

This case serves as a warning of the risk of further spread and the risk of outbreaks in the United States. Clues to diagnosis of OROV are travel to an endemic region; prominent headache with fever, chills, fatigue, malaise, myalgias, arthralgias, back pain, abdominal pain, diarrhea, nausea, vomiting, maculopapular rash, photophobia, retro-orbital pain, conjunctival injection, mucosal bleeding, and biphasic illness; and, in the case of neuroinvasive disease, symptoms suggestive of aseptic meningitis (eg, photophobia, dizziness, retroorbital eye pain, and neck stiffness) [10]. This case also illustrates the critical importance of collaboration between state laboratories and the CDC. The Wisconsin Department of Health Services contacted our team, allowing us to relay the results to the patient in the clinic.

## Note

***Potential conflicts of interest.*** All authors: No reported conflicts.
